# Supplementary material for: Content-rich biological network constructed by mining PubMed abstracts
Source: BMC Bioinformatics. 2004 Oct 8;5:147. doi: 10.1186/1471-2105-5-147 (PMC528731; doi:10.1186/1471-2105-5-147)
Supplement: Additional File 2 — The original results of the above study (non-essential files are deleted to keep the file size under the limit set by BMC bioinformatics). [file 1471-2105-5-147-S2.bz2 › chilibotAdditionalFile2/dip05/602ID9751050E10981/html/CDK6_CDKN2A.html]

 


 **CDK6** and **CDKN2A** 
  
Found 170 abstracts in PubMed, retrieved 05.  
 

 What does Google say? 
 PDF only 
| .edu only 

---

**Interactive relationship** (e.g. stimulation, inhibition, etc)

**Inhibitory relationship**- The INK4  [ **CDKN2A** ]  family of cyclin dependent kinase CDK inhibitors negatively regulates cyclin D dependent CDK4 and  **CDK6**  and thereby retains the growth suppressive function of Rb family proteins.  Ref: 12556487 Mol Cell Biol, 2003
**Neutral relationship**

**Non-interactive relationship** (e.g. studied together, co-existance, homology, etc.)

- As shown by immunoblot analysis, EGCG treatment of LNCaP and DU145 cells resulted in significant dose and time dependent i upregulation of the protein expression of WAF1 p21, KIP1 p27, INK4a  [ **CDKN2A** ]  p16, and INK4c p18, ii down modulation of the protein expression of cyclin D1, cyclin E, cdk2, cdk4, and  **cdk6** , but not of cyclin D2, iii increase in the binding of cyclin D1 toward WAF1 p21 and KIP1 p27, and iv decrease in the binding of cyclin E toward cdk2.  Ref: 12559991 Arch Biochem Biophys, 2003
- Transition through this point requires  **cdk6**  4 cyclin D, since inhibition with TAT p16 INK4A  [ **CDKN2A** ]  during the first 3 to 5 h prevents cell cycle entry and maintains both naive and memory T cells in G 0.  Ref: 12640120 Mol Cell Biol, 2003
- Components upstream of the retinoblastoma tumour suppressor pRB, namely the INK4  [ **CDKN2A** ]  family of cyclin dependent kinase CDK inhibitors, the D type cyclins, their partner kinases CDK4  **CDK6** , and pRB as their critical substrate, are differentially targeted in diverse types of cancer.  Ref: 12754735 J Pathol< MedlineTA>J Pathol, 2003
